# Study of the effect on shelter cat intakes and euthanasia from a shelter neuter return project of 10,080 cats from March 2010 to June 2014

**DOI:** 10.7717/peerj.646

**Published:** 2014-10-30

**Authors:** Karen L. Johnson, Jon Cicirelli

**Affiliations:** 1 National Pet Alliance, San Jose, CA, United States; 2 San Jose Animal Care and Services, San Jose, CA, United States

**Keywords:** Shelter, Trap, Neuter, Return, Feral cat, Population control, Cat euthanasia, Population policy

## Abstract

Cat impoundments were increasing at the municipal San Jose animal shelter in 2009, despite long-term successful low cost sterilization programs and attempts to lower the euthanasia rate of treatable-rehabilitatable impounds beginning in 2008. San Jose Animal Care and Services implemented a new strategy designed to control overall feral cat reproduction by altering and returning feral cats entering the shelter system, rather than euthanizing the cats. The purpose of this case study was to determine how the program affected the shelter cat intakes over time. In just over four years, 10,080 individual healthy adult feral cats, out of 11,423 impounded at the shelter during this time frame, were altered and returned to their site of capture. Included in the 11,423 cats were 862 cats impounded from one to four additional times for a total of 958 (9.5%) recaptures of the previously altered 10,080 cats. The remaining 385 healthy feral cats were euthanized at the shelter from March 2010 to June 2014. Four years into the program, researchers observed cat and kitten impounds decreased 29.1%; euthanasia decreased from over 70% of intakes in 2009, to 23% in 2014. Euthanasia in the shelter for Upper Respiratory Disease decreased 99%; dead cat pick up off the streets declined 20%. Dog impounds did not similarly decline over the four years. No other laws or program changes were implemented since the beginning of the program.

## Introduction

In an ongoing effort to find a solution to the problem of too many cats entering local shelters and not leaving alive, two major programs were started in San Jose, and surrounding areas. The goal was to reduce the intact cat populations.

The first was the free Spay/Neuter Voucher Program, initiated and funded by the City of San Jose in 1994 (Benninger, 1995). This program encompassed any cat, whether owned or unowned. This program is no longer free.

The second program began in March 2010 and is ongoing, targeting only feral cats impounded at the shelter (County of Santa Clara, 2011). These cats, even when healthy, are not suited for adoption and therefore euthanasia would typically be their fate. The goals of the program were to reduce euthanasia of healthy cats, even if feral, reduce the costs to handle feral cats by animal services by reducing intake, and to reduce the reproductive ability of feral cats in the community.

This is a continuation of efforts to measure how Animal Control programs can affect the differing segments of cat populations in the county.

## Background

In conjunction with the start of the economic recession in 2007–2008, San Jose Animal Care and Services (SJACS) cat impounds substantially increased by nearly 1,000 cats in just one year. More than 70% of the adult cats entering the shelter were being euthanized. Local cat advocates discussed a program with the shelter manager, based on a 2008 Jacksonville, Florida, USA plan (Mitchell, 2008), to alter all healthy feral cats impounded at the shelter, and then return the cats to their place of capture, rather than euthanize them as healthy but unadoptable. This was viewed as a controversial program, as some groups believe feral cats are killing too many birds (Loss, Will & Marra, 2013), or live short, brutal lives (Jessup, 2004). The program began as a pilot in March 2010. Initially the program was called Feral Freedom, and is now referred to as Shelter Neuter Return (SNR).

Santa Clara County, California is a large 1291 square mile area containing an estimated 1,868,558 people in 2014. San Jose is the largest city in Santa Clara County, with an estimated population of 1,000,536 people as of 2014 (California Department of Finance, 2014). The county is located at the southern end of the San Francisco Bay Area in Northern California.

The San Jose shelter has animal control service contracts with Cupertino, Los Gatos, Milpitas and Saratoga. The five cities or towns service area contains 63% of the county population (US Census Bureau, 2012; California Department of Finance, 2014; City of San Jose, 2014). This shelter also investigates cruelty complaints for their service area.

A random 1,000 household study of the county dog and cat population was conducted in 1993 (Cat Fanciers’ Almanac, 1994) and repeated in 2005 (Kass, Johnson & Weng, 2013) by a local non-profit organization. The major finding in the 1993 study was 41% of the known estimated cat population were unowned cats. These community cats were fed, but not claimed to be owned by the respondents. Additionally, in 1993, 86% of owned cats were reported altered, but 16% had a litter prior to altering. The number of unfed community or feral cats was, and remains, unknown.

In response to these findings, in 1994, the City of San Jose began the highly successful, free spay/neuter program in order to stem the increasing cat intakes at the shelter, and reduce the number of owned and unowned reproducing cats. Santa Clara County implemented a similar program five years later (County of Santa Clara, 2011). There have been changes in funding and qualifications for the programs over the years. The (formerly) free feral cat altering program is now $25 in San Jose and includes vaccinations against rabies and other common cat disease, flea treatment, ear treatment, microchip, and ear tipping (San Jose Animal Care and Services, 2014).

Two major, and five smaller, shelters operate within Santa Clara County. The Humane Society Silicon Valley (HSSV), which accepted nearly 25,000 cats at its peak in 1990, and San Jose Animal Care and Services (SJACS), which opened in 2004, are the two largest.

The small shelter in San Martin, operated by the County of Santa Clara (SCCo), serves primarily the rural unincorporated portions of the county. Silicon Valley Animal Control Authority (SVACA) operates in the City of Santa Clara, servicing four cities with a combined population of 243,453 (13%) of the county population (US Census Bureau, 2012).

A small government shelter operates in the Palo Alto area. Their data was not included in the two 1,000 household studies due to lack of information at the time. However, as of 2007, the Asilomar reports from Palo Alto Animal Services (PAAS) were published, and this agency is now included in the overall county shelter data. The PAAS service area is Palo Alto, Los Altos, and Los Altos Hills, containing 101,365 (5.7%) of the county population (US Census Bureau, 2012).

HSSV handled the majority of the animal control and investigative services for the entire county until the San Jose and SVACA shelters opened, and now HSSV provides animal sheltering services only for the City of Sunnyvale (B Ward, pers. comm., 2014). HSSV now handles about 4,000 animals per year in their new Milpitas facility (Humane Society Silicon Valley, 2013).

In addition, Town Cats (TC) and Palo Alto Humane Society (PAHS) are two small privately run shelters that are not government entities, and perform no cruelty investigations.

Six shelters in the county are working together to permanently end the euthanasia of every adoptable animal impounded. This coalition of animal shelters is known as We CARE and adheres to Maddie’s Fund statistical reporting and requirements to not euthanize any healthy adoptable animals. This is accomplished by communicating and transferring animals between the various shelters based on capacity and need. The shelters are very close to ending the euthanasia of all treatable-rehabilitatable impounded animals in the county as indicated in the last published Maddie’s Fund report from 2011, which shows only 6% of cats euthanized for the entire county were treatable-rehabilitatable (Maddie’s Fund, 2011). This project began informally in 2008 with a Memo of Understanding signed in 2011 (Santa Clara County, 2011).

The shelters can be further defined as either “source” or “receiving” shelters. All the government shelters are sources of animals. HSSV, TC, PAHS, and many other rescue groups, receive animals from the government shelters. In the event of a large contagious situation at one shelter, other shelters may transfer to their shelter the remaining healthy animals, until such time as the disease in the affected shelter is under control. Resources, such as large food donations, are shared, along with the expense of multiple combined advertising promotions each year in accordance with the Memo.

A steady decrease and leveling off in shelter cat intakes was observed until 2008 when just over 14,000 cats entered the shelters countywide. This 50% drop in cat impoundments from 1990–2008 has been largely attributed to the spay/neuter voucher program (Kass, Johnson & Weng, 2013) and volunteers altering feral cats.

A follow up 1,000 household study of the pet population in 2005 found 93% of owned cats in Santa Clara County were reported by their owners to have had alteration surgery, rather than the prior 1993 rate of 86%. However, unowned but fed cats were reported altered by their caregivers at only a 5.5% rate. Further, in 1993, 16% of owned cats had a litter prior to being altered, while in 2005, less than 0.5% of owned cats were reported to have had a litter prior to being altered (Kass, Johnson & Weng, 2013). The vast majority of owned cats are reported altered, yet many cats and kittens are still being impounded as free roaming stray cats at the local shelters and almost all of them are unaltered. In 2013, only 2% of cats were reclaimed by owners in San Jose (170 out of 8,643), suggesting a low level of ownership among the shelter population. It seems apparent that the only substantial cat population left to alter would be the feral population.

The goal of this report is to show the changes in cat intakes and euthanasia at the San Jose shelter from fiscal years 2010 to 2014 during the SNR program, and how the impounds differed among the five county shelters reporting data overall. Prior declines in the county shelters intakes as a result of the free and low cost alteration programs had leveled off. A fresh approach was needed to further reduce shelter cat impounds and euthanasia.

## Methodology

Shelter data was provided to, and maintained by, the authors on an annual fiscal basis from program managers, shelter officials, or Board reports for each shelter listed. The calendar year annual shelter reports were generally not publicly available until 2007, when Asilomar Accords (Maddie’s Fund, 2011) reporting standards were adopted by five of the six local shelters whose data is included in this report.

Data provided by the shelters did not include owner requested euthanasia, quarantine, or dead on arrival cats. Fiscal year (FY) data is provided from July 1st to June 30th of each year. Calendar year (CY) data is based on January to December of each year.

City of San Jose, State of California Department of Finance, and US Census Bureau population data was used to determine the human populations of individual cities in the county, which was then used to estimate the number of cats handled per 1,000 humans at the shelters.

The euthanasia rate is calculated by dividing the number of cats euthanized by total live intake, less owner requested euthanasia, and dead on arrival, in accordance with Maddie’s Fund/Asilomar reporting requirements (Maddie’s Fund, 2011). The Save Rate is calculated by subtracting the euthanasia rate from 100. The euthanasia rate does not include those cats who died of natural causes while in the shelter, which is 1% or less annually.

The program requires incoming feral/free roaming cats be held 72 h, and these cats are included in shelter intake numbers. Within 24 h of entering the shelter system the cats are evaluated for inclusion in the SNR program. All cats are scanned for a microchip, however, due to the difficulty of handling feral/fractious cats, most are not scanned thoroughly for a microchip until they are sedated and prepared for surgery. Once the shelter identifies a cat that is healthy, but fractious enough to exclude from the adoption program, it is scheduled for alteration surgery and medical services as quickly as possible, and ideally while the holding period is still in effect. The objective is to move cats out of the shelter system as rapidly as possible.

If deemed behaviorally unadoptable and suitably healthy, the cats have the alteration surgery, ear tip removal for identification in the field after return, rabies and common cat disease vaccinations, microchip, and a recovery period at the shelter during the state mandated 72 h hold. Flea/tick treatment, ear cleaning and other minor medical issues, such as abrasions, are also addressed as indicated. Once the cats have recovered from surgery, medical staff approve the cats for release to a non-profit, pursuant to California Food and Agricultural Code 31752, which states in the relevant part: “Any stray cat shall be released to a nonprofit, if requested, prior to the euthanasia of the cat”. This non-profit group then returns the cat to their capture site.

The criteria to establish whether or not to include a cat in the SNR program are as follows:

Friendly (passes behavior)

• Approaches handler
• Solicits attention
• Easy to handle and seems to like handling
• Seeks further attention when handler stops interacting

Shy (passes behavior)

• Does not approach
• May hiss initially
• May avoid handling at first by backing away, becoming stiff
• Once handled, solicits attention like the friendly cat
• Becomes easy to handle
• Seeks further attention when handler stops interacting
• No longer hisses

Fearful (SNR - rescue or hold longer to see if can become shy)

• Does not approach
• May hiss initially
• Avoids by running away or becoming stiff, may show whale eye
• Able to handle, but remains stiff, may clutch onto bars, hands, arms
• Scampers away after handling
• May continue to hiss

Feral/fractious (SNR)

• Does not approach
• May hiss and strike
• Frantically darts about the cage, or may stiffen in place
• Not able to get hands on due to striking, lunging, trying to bite

There is no upper age limit for a cat to be included in the program provided it is healthy. Kittens may be included in the program at four months of age, or less, if there is an identified caretaker or colony manger, or if the queen is present.

Citizens who are turning over trapped cats to the shelter sign a release form that acknowledges SNR, euthanasia, and adoption as any of the possible outcomes for the cat. Cats are not returned to their trapped location in high impact situations, such as hoarding, dangerous conditions, or officially designated wildlife protection areas. When cats are returned to the capture site, door hangers are placed in proximity to the returned cat, advising the neighbors of the program. If a feral cat cannot be returned to the site of capture, it may still be euthanized. This program does not support the relocation of cats to new areas.

In the event the cats are a problem to the neighborhood, and there is objection to the cat being returned, mediation is attempted, though not always successfully, through shelter volunteers, rescue partners, and staff, including enforcement when needed.

## Results

### Santa Clara county

From l982–1990 shelter cat impounds increased 29%. In 1990 nearly 28,000 cats entered the HSSV and SCCo shelters. Cat intakes were 18.7 per 1,000 humans in 1990 for the county, but by the end of fiscal year (FY) 2014, intakes were 7.0 per 1,000 humans.

**Figure 1:**
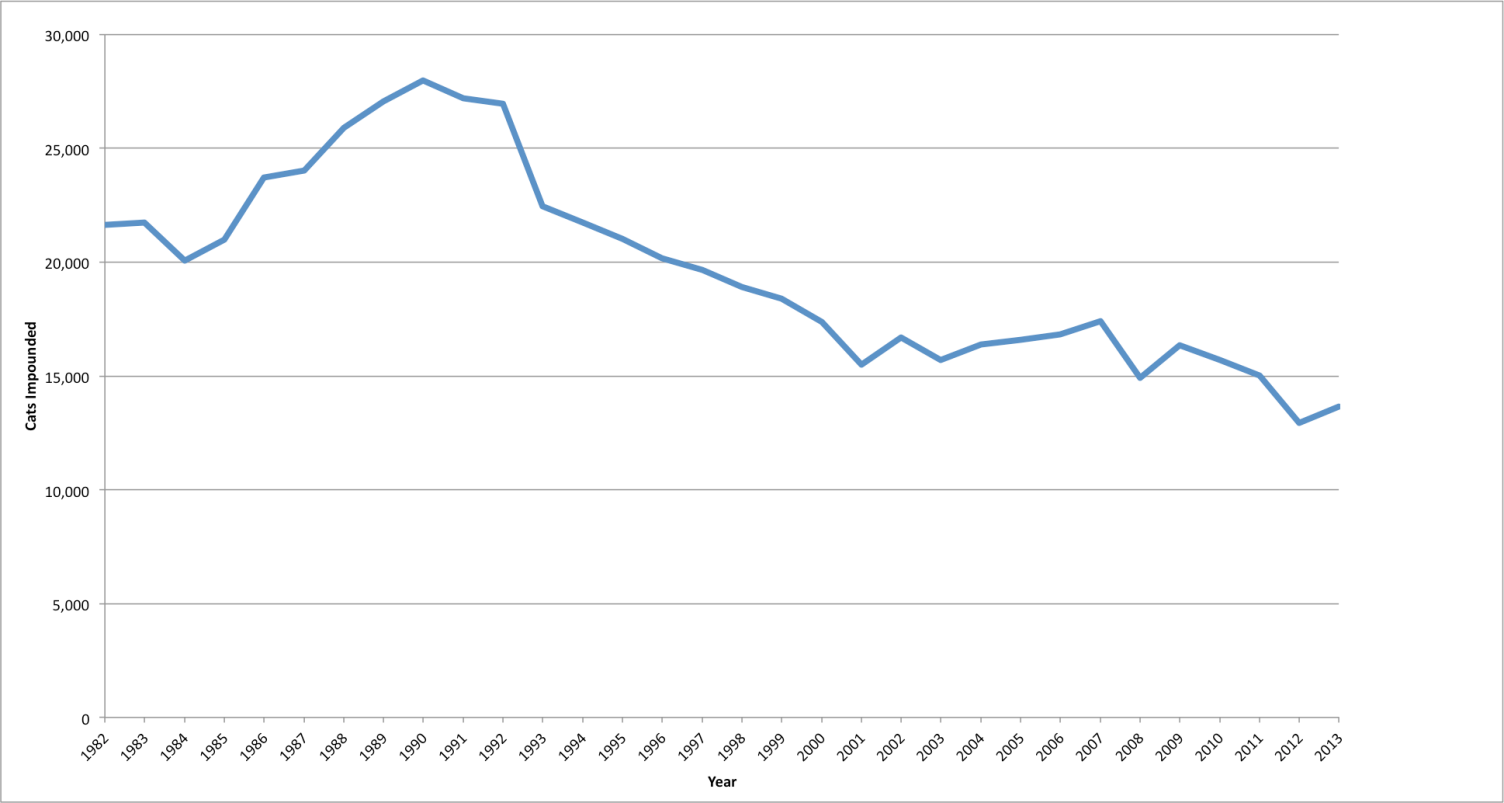
Santa Clara county cat intakes from five shelters since 1982. Available annual basis shelter cat intake records are grouped together here from the five Santa Clara county shelters providing data.

Figure 1 shows the cat intakes from five county shelters since 1982 (KL Johnson, 2014, unpublished data). Palo Alto information is only included as of 2007 (City of Palo Alto, 2013). The data do not include cats dead on arrival, cats with owner euthanasia requests, or cats that naturally died in the shelter.

Figure 1 shows available cat intake numbers encompassing the combined five shelters. There is a long steady decline in cat intakes coinciding with the start of the San Jose Free Spay/Neuter Program. The intakes then reached a plateau until the beginning of the SNR project in 2010. The substantial 29.1% decrease in cat impounds from 2009 is dramatic, and at the lowest impound levels since 1982 impound records were kept.

Table 1 shows the exact intake for each shelter. San Jose opened in 2004, SVACA opened in 2007, and PAAS had no publicly available information prior to 2007.

**Table 1:** Annual cat impounds at five Santa Clara county shelters. Raw data showing annual cat impounds at the five Santa Clara county shelters providing data from 1982–2013.

Year	SCCO	HSSV	SJACS	SVACA	PAAS	Annual total
**1982**	2,114	19,537				**21,651**
**1983**	2,219	19,534				**21,753**
**1984**	2,236	17,838				**20,074**
**1985**	2,117	18,868				**20,985**
**1986**	2,653	21,075				**23,728**
**1987**	2,834	21,185				**24,019**
**1988**	3,425	22,476				**25,901**
**1989**	3,620	23,431				**27,051**
**1990**	3,360	24,637				**27,997**
**1991**	3,137	24,077				**27,214**
**1992**	3,640	23,309				**26,949**
**1993**	2,830	19,643				**22,473**
**1994**	1,715	20,021				**21,736**
**1995**	1,656	19,377				**21,033**
**1996**	1,488	18,668				**20,156**
**1997**	1,477	18,184				**19,661**
**1998**	1,591	17,329				**18,920**
**1999**	1,274	17,106				**18,380**
**2000**	1,108	16,281				**17,389**
**2001**	1,646	13,843				**15,489**
**2002**	1,351	15,349				**16,700**
**2003**	1,299	14,419				**15,718**
**2004**	1,504	14,865				**16,369**
**2005**	1,632	7,812	7,152			**16,596**
**2006**	1,646	4,349	10,825			**16,820**
**2007**	2,007	3,543	10,777	577	498	**17,402**
**2008**	1,628	1,741	10,003	1,066	486	**14,924**
**2009**	1,695	1,647	11,429	1,096	489	**16,356**
**2010**	1,660	2,288	10,465	801	484	**15,698**
**2011**	1,428	2,705	9,558	922	399	**15,012**
**2012**	1,207	1,908	8,545	868	410	**12,938**
**2013**	1,512	2,085	8,879	870	316	**13,662**

**Figure 2:**
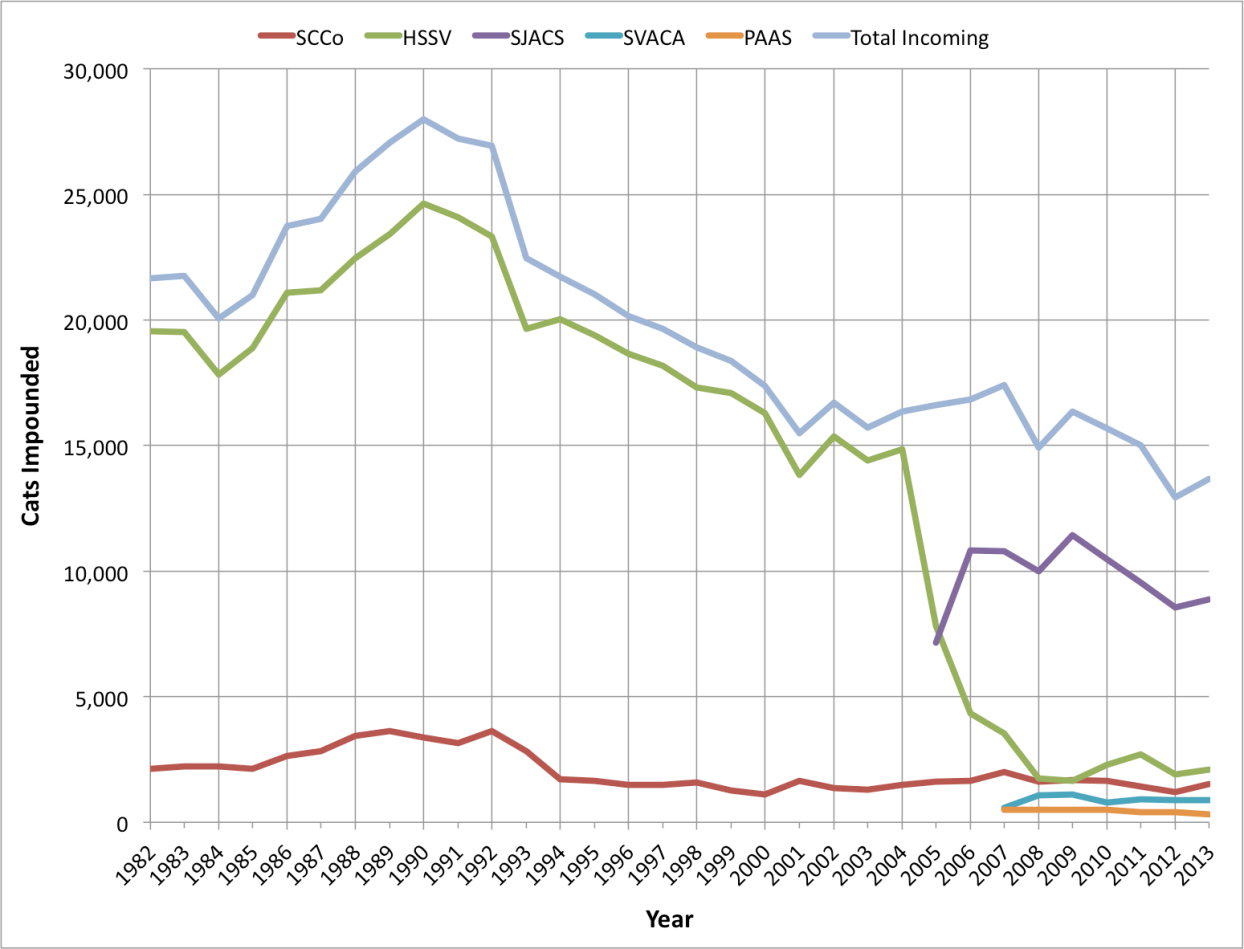
Annual shelter cat intakes are shown individually from the five Santa Clara county shelters providing data.

The information from Table 1 is also shown on Fig. 2, giving a visual reference to how the cat intakes changed over time as animal control contracts were absorbed by the new shelters.

The difference in cat intakes among three of the shelters from 2009 to 2013 do not show the same magnitude of decrease as SJACS, and in the case of HSSV, increased by 438 cats. SVACA also began the SNR program in 2010 (D Soszynski, pers. comm., 2014), and produced a 21% decrease in intakes as of 2013 from their high of 1,096 in 2009.

### San Jose

Expenses to alter the feral cats were approximately $72 per cat; however, the annual stray cat population in the shelter has decreased by more than 3,000 cats per year, resulting in fewer expenditures for cats that require housing, euthanasia, cleaning chemicals and all the various materials, supplies and personnel needed to care for those cats. Expense to return cats to their capture site was handled by the rescue volunteers.

A total of 10,080 individual feral cats and kittens were impounded at the shelter, processed through the SNR program, and returned back to their site of capture from March 2010 until June 2014. As a result of the ear tip removal and microchip identification, 862 additional cats were identified as already processed through the program from one to four times, for a total of 958 (9.5%) subsequent repeat impounds (Table 2).

**Table 2:** Recaptured SNR cats at SJACS, 2010–2014. SNR cats were recaptured for one to four additional roundtrips to SJACS.

Additional	No. of cats	Total additional
**Captures**		**Impounds**
1	782	782
2	68	136
3	8	24
4	4	16
	862	958

Altogether, there were a total of 11,038 impounds and returns, including the 958 subsequent recaptures.

The shelter received again 185 (1.8%) dead on arrival, of the 10,080 SNR cats, identified by microchip, who had previously been through the SNR program. The cause of death was not specified in the records. Dead on arrival cats were not included in the additional impound data.

From March 2010 to June 2014, 385 cats (3.4%) were euthanized as a healthy feral, but unadoptable, out of the total 11,423 healthy feral cats impounded alive at the shelter. Most of the euthanasia of healthy feral cats was primarily done at the beginning of the SNR program, in 2010, while the staff was still determining some of the program parameters. As of June 2014, only 13 feral cats were euthanized healthy in the prior 12 months. If the cat was sick or injured, the cat was categorized “euthanized-sick/injured” rather than feral. When a cat was euthanized as a healthy feral, it was because the location to return the cat was not deemed safe or viable. This can occur when the location is directly adjacent to a freeway or a location where there was hoarding and there are too many cats to return to one neighborhood location. As a general rule, the shelter does not support the relocation of feral cats to new areas.

In CY 2009, the San Jose shelter euthanized 8,466 cats, including 252 owner requested euthanasia. Data from their Asilomar report indicates 6,651 cats were euthanized as unhealthy and untreatable (San Jose Animal Care and Services, 2009). By the end of FY 2014, total cat euthanasia, including 111 owner requested euthanasia, declined to 1,954 of which 1,504 cats were determined to be unhealthy and untreatable.

Additionally, it was found the number of dead cats picked up on the street declined 20% from 1,629 in CY 2009 to 1,308 in FY 2014, and the number of cats euthanized for Upper Respiratory Infection (URI) in the shelter declined from 736 in CY 2009, to seven in FY 2014, or a 99% decrease.

**Figure 3:**
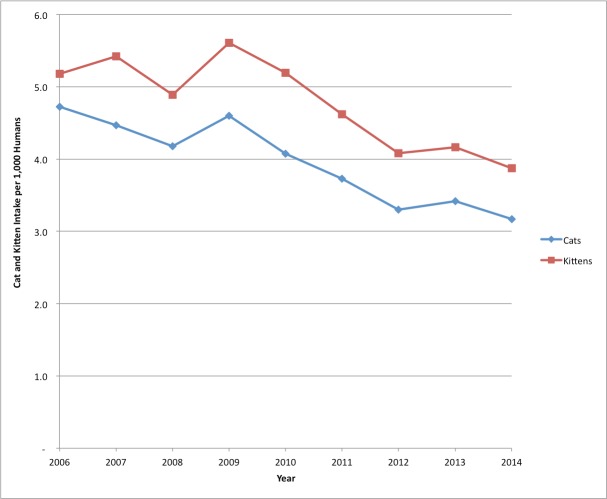
SJACS cat impounds 2006–2014 per 1,000 humans. SJACS total shelter cat intakes from 2006–2014, including cats entering from contract cities, in comparison to the human population.

Santa Clara County’s human population has increased by over 300,000 individuals since 1990 (California Department of Finance, 2014). To better understand the cat intakes in comparison to the human population, Fig. 3 shows the adult cats and kittens handled through the San Jose shelter in comparison to 1,000 humans for the time period 2006–2014. As the SJACS animal control contracts include Saratoga, Los Gatos, Milpitas and Cupertino, the following figure includes the total combined human population of all the contract cities.

The decline in adult cat and kitten intakes at the shelter is readily apparent, coinciding with the implementation of the SNR program. In 2009, when cat intakes were climbing, total impoundment for cats and kittens was 10.2 per 1,000 humans. By 2014, after four years of SNR, the total cat impound rate had decreased to 7.0 per 1,000 humans, or a total of 29.1% fewer cat impounds.

**Figure 4:**
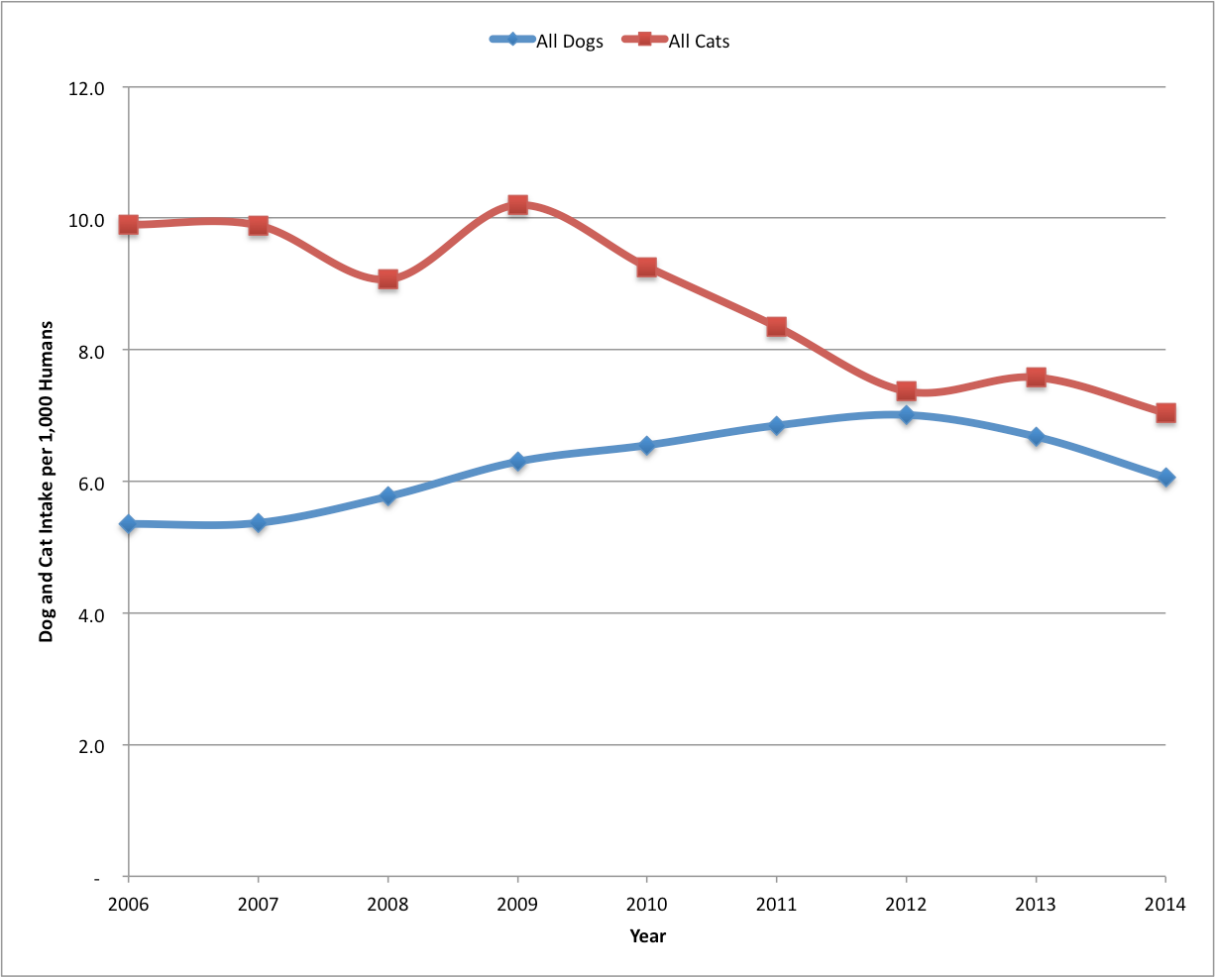
SJACS dog and cat impounds 2006–2014. Comparison of annual dog and cat intakes at SJACS from 2006–2014, per 1,000 human population.

For comparison purposes, Fig. 4 showing both dog and cat intakes per 1,000 humans is shown below.

**Figure 5:**
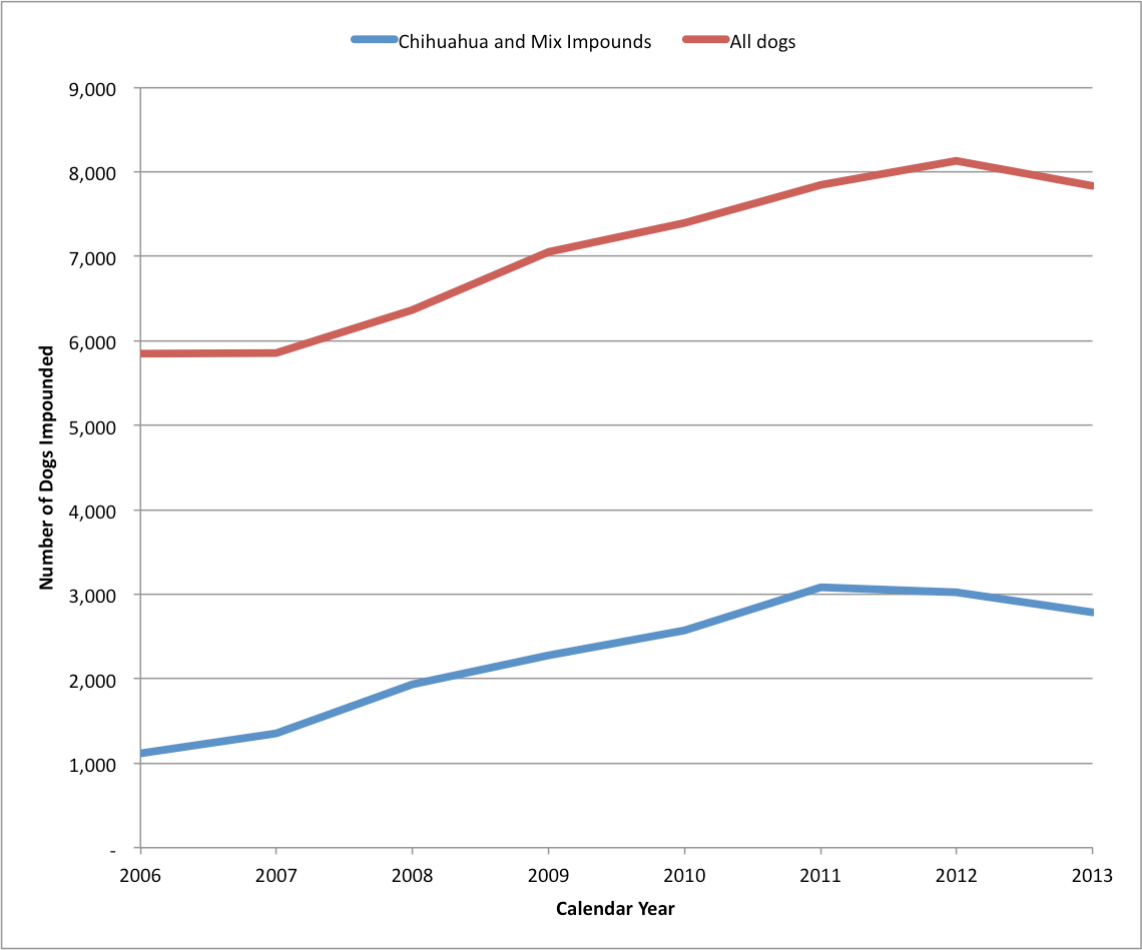
SJACS total dog impounds 2006–2013. SJACS annual dog impounds, showing how Chihuahuas and mixes of that breed contribute to the shelter intakes, from 2006–2013, on a calendar year basis.

Dog impounds were increasing until 2011 when San Jose also began a targeted free Chihuahua altering program through a grant with Petsmart Charities and in partnership with the Humane Society Silicon Valley. Prior to the grant program for dogs, their intake was increasing steadily each year. The dog impound increase was primarily driven by Chihuahuas, and mixes of that breed, which made up almost 40% of total dog intake at the time the dog program began. Figure 5 is shown in a calendar year basis.

Dog intakes began decreasing in 2012. In 2014, there were 6.1 dogs impounded per 1,000 humans, down from 7.0 in 2012. Cats were impounded at 7.0 per 1,000 humans. In 2006 there were 5.4 dogs and 9.9 cats impounded per 1,000 humans.

**Figure 6:**
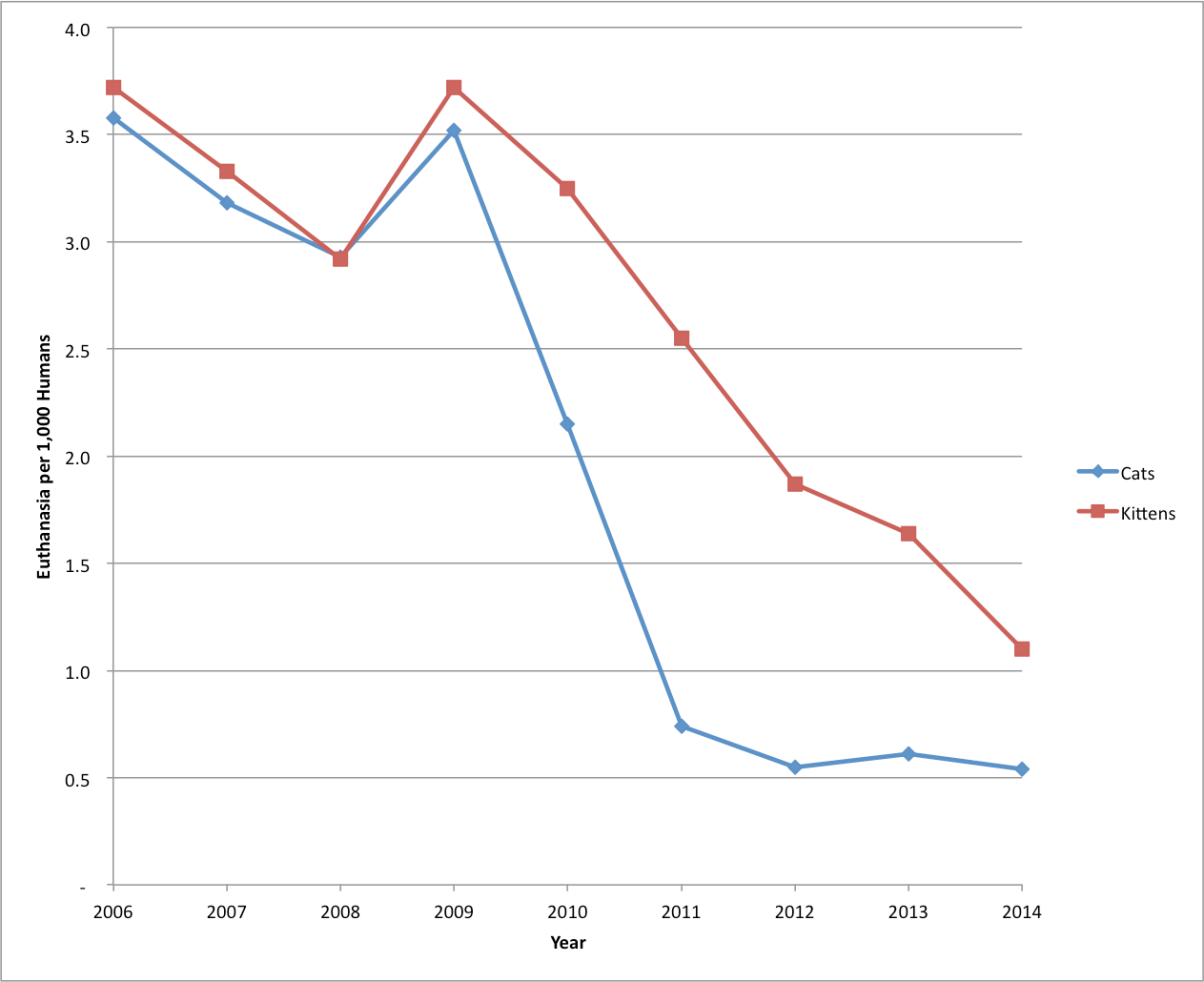
Cat Euthanasia at SJACS 2006–2014. Annual euthanasia of impounded cats at SJACS from 2006–2014 per 1,000 humans in San Jose and the contracted cities.

The euthanasia rate was expected to drop, at a minimum by the number of feral cats returned, rather than euthanized. Figure 6 shows the euthanasia rate per 1,000 humans for adult cats and kittens separately.

Adult cat euthanasia in 2006 was 3.6 per 1,000 humans while kitten euthanasia was 3.7 per 1,000 humans. During the 2009 spike year 4,163 kittens, and 3,943 cats were euthanized. As of 2014, 713 adult cats and 1,917 kittens were euthanized, for a rate of .5 per 1,000 humans for adult cats, and 1.1 for kittens.

**Figure 7:**
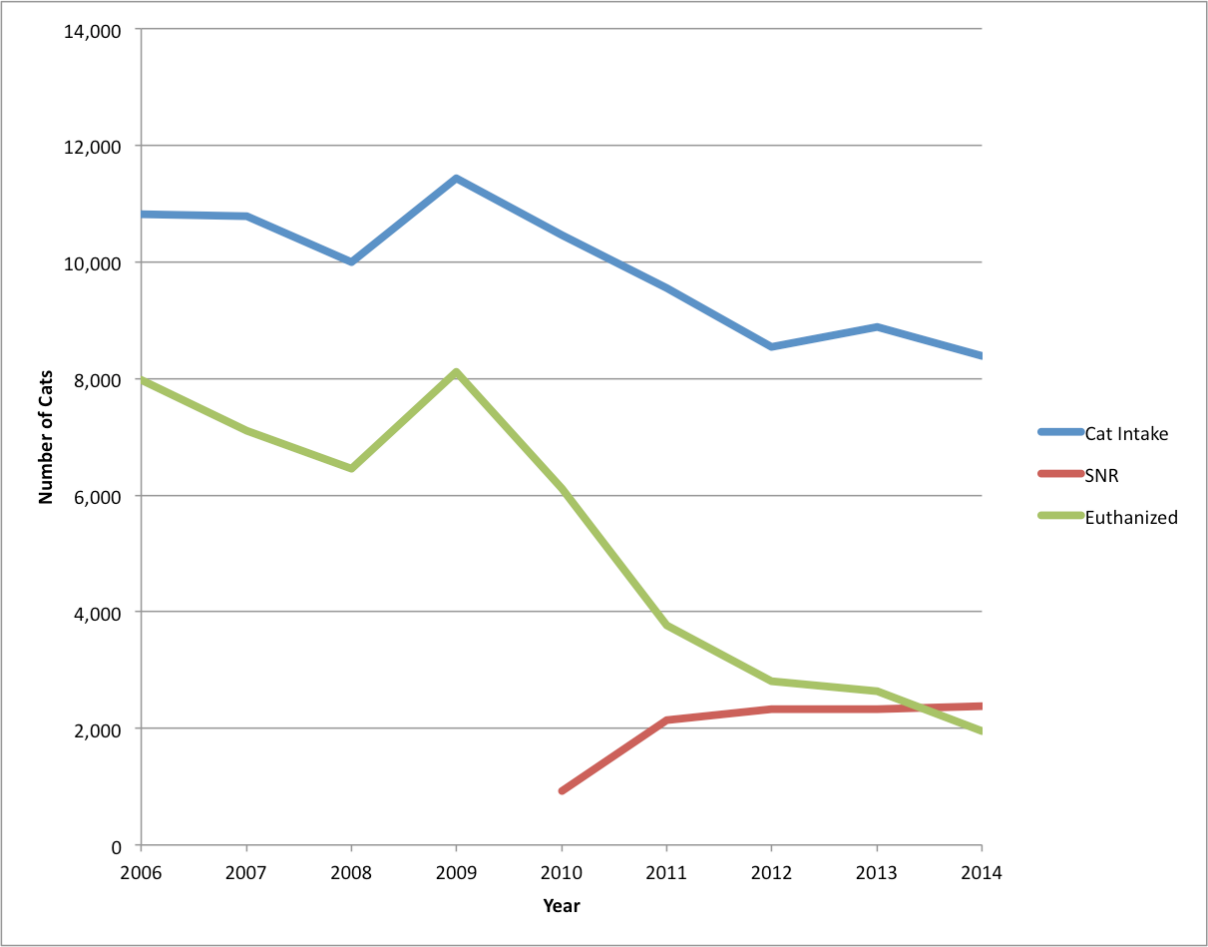
Comparison of SJACS cat intake, euthanasia and SNR cats, 2006–2014. Annual cat intake, 2006–2014, at SJACS compared with euthanasia and the number of SNR feral cats returned.

Figure 7 combines total cat and kitten intake, total euthanized, and number of SNR cats, and shows how altering and returning an average of 2,328 feral cats annually for 4.3 years, affected both intakes and euthanasia. The 2014 annual cat intake was 3,043 less than the 11,429 cat intakes in 2009. Annual cat euthanasia was 6,152 less than 2009.

**Figure 8:**
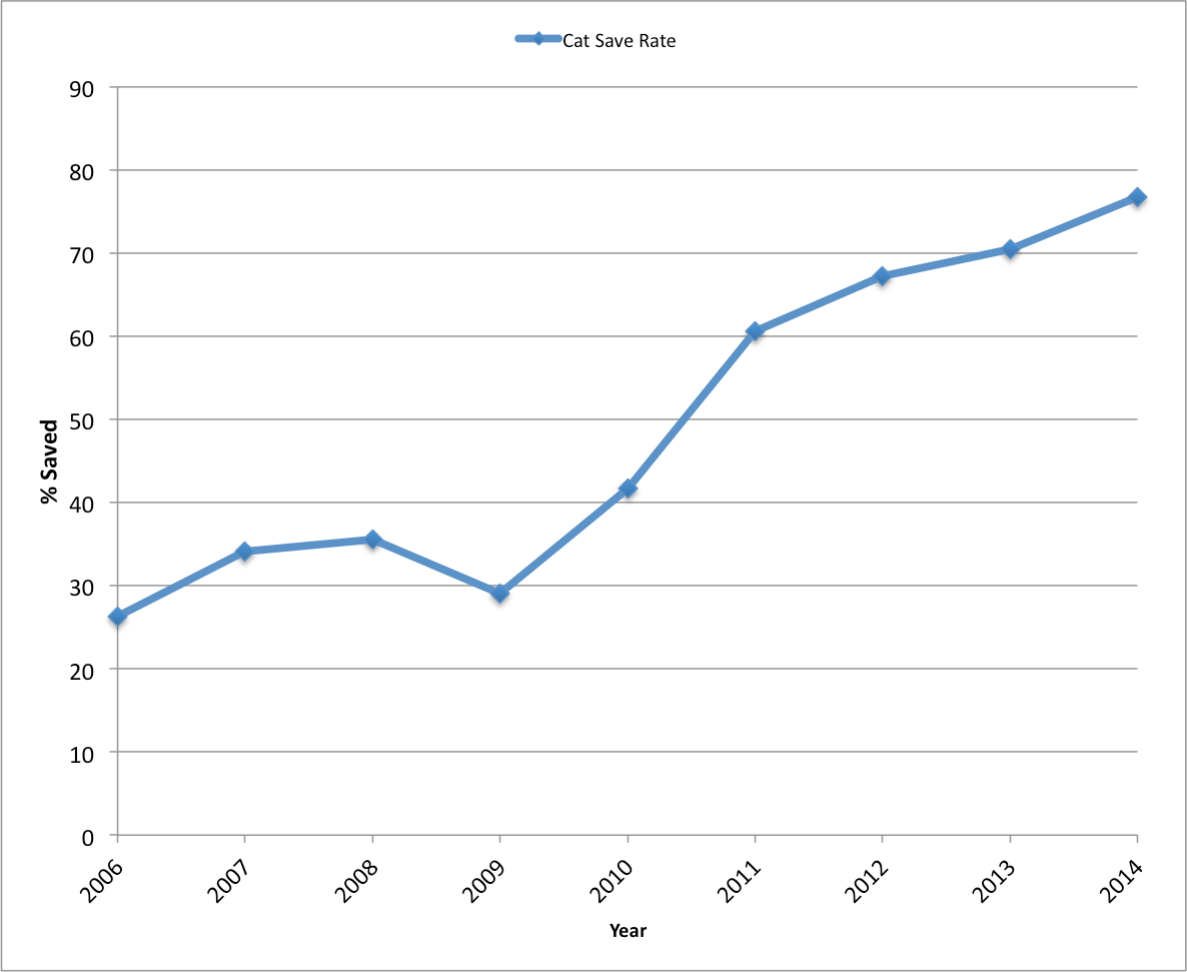
SJACS cat save rate, 2006–2014. Percent of cats saved at SJACS from 2006–2014.

The save rate of shelter cats and kittens has increased markedly, as demonstrated in Fig. 8. The data does not include cats and kittens euthanized per owner request, dead on arrival, or died in the shelter of natural causes. The combined cat/kitten save rate increased from 29.1% in 2009, to 76.7% in 2014.

In Santa Clara County three additional shelters have now started their own SNR programs—SVACA in 2010 (D Sozynski, pers. comm., 2014), SCCo in 2013 (A Escobar, pers. comm., 2014), and HSSV in 2014 (B Ward, pers. comm., 2014). SVACA (Silicon Valley Animal Control Authority, 2014) data, comparing 2009 cat intakes to 2013, showed a 21% decrease in cat impounds, similar to SJACS results from the SNR program.

## Discussion

No attempts to determine the fate of the cats released back to their site of capture were made. With the decline of dead cats picked up off the street, the outdoor cat population is either diminishing further, or it is possible that fewer surgically sterilized cats are roaming wide areas due to lack of mating interest. In one project (Loyd et al., 2013), researchers found 45% of roaming cats crossed roads in 7 days. In yet another report, caregivers of feral cats reported decreased tendency to roam after being neutered (Scott et al., 2002).

Recaptured cats encompassed 9.5% of the feral cats altered through the program. The ear tip removal for identification should alert the trapper to the alter status of the cat. Further education of individuals trapping cats to be on the lookout for the ear alteration is necessary, to avoid wasting resources. The microchip and ear tip removal did successfully alert the shelter staff, and prevented needless surgery.

The SNR cats are viewed as “community cats”. It is highly likely they were born in the neighborhood they came from, and have lived most or all of their life there. These impounded feral cats are surprisingly healthy and have good bodyweight. Their good health and body condition guides the decision of shelter medical staff whether or not to include them in the SNR program.

The two 1,000 household studies in Santa Clara County show the known fed, yet unowned, cat population declined from 41% of the known cat population (Cat Fanciers’ Almanac, 1994) to 35% by 2005, while the owned cat population decreased from 30% of households to 25% (Kass, Johnson & Weng, 2013). It is apparent the cat population, both owned and unowned, diminished over the 12 years between the two studies.

The impact of not having to care for more than 3,000 additional cats annually allows staff and management to focus on other areas of the operation and pursue other welfare related strategies. The internal capacity of the organization to help other animals is increased without requiring more staff.

It was expected the number of cats euthanized would drop, simply due to the feral cats being returned to the capture site rather than euthanized. No other program changes or laws were implemented during this project. The 99% decrease in euthanasia for URI in the shelter was an additional positive result indicating better shelter capacity to help these cats. Large volumes of cats in any shelter system elevate the risk of disease acquisition and transfer (Pedersen et al., 2004). URI is one of the most prevalent cat health concerns facing shelter personnel. Feral and fractious cats experience extreme stress in the shelter environment, making all cats more susceptible to URI. “Because of its close association with herpesviral activation and stress, URI is also a bellwether for overall shelter cat health and wellbeing” (UC Davis).

At a 2011 conference (Cicirelli & DuCharme, 2011) data from the 2008 Jacksonville, Florida, USA shelter neuter return project, known as Feral Freedom, showed a 23% decrease in cat intakes after 3 years from their SNR program. Similar results have also been produced with this type of program in Albuquerque, New Mexico, USA (Sizemore, 2014) where a 22% drop in kitten intakes was observed after 2 years. The SVACA 21% decrease in cat impounds after three years of SNR were similar to SJACS, giving corroboration to the data received.

This case study demonstrates the positive impacts of alteration surgery and return of feral cats in a community, preventing the proliferation of feral cat reproduction flow into the community and the shelters. Reduction of cat impounds results in a long-term economic benefit to municipalities when less animal control expenditures are required for a jurisdiction. The primary goals of an SNR program are to humanely reduce outdoor cat populations, shelter cat intake, and cat euthanasia. If achieved, these reductions were expected to produce overall savings. Ceasing this program would likely have alarming implications.

## Study Limitations

It is unknown if the economic recession was a contributing factor to the sudden increase in cat impoundments in San Jose. Certainly caregivers of the feral cats could have decided to reduce their cat feeding expenses during the recession. Of the five shelters reporting cat impound data for 2009, only San Jose showed a large increase. Countywide, a total of 1,432 additional cats were impounded at the five shelters from the previous year, of which 1,426 were attributed to the San Jose shelter.

There was no follow up on the welfare or status of the returned feral cats, other than the 9.5% recaptures and the 2% received dead on arrival at SJACS.

No scientific analysis has been made, as this report is intended to be purely descriptive of changes in cat intake and euthanasia at the San Jose shelter since the inception of the SNR program.

The current estimated owned and unowned cat populations are unknown.

## Conclusion

The SNR program is recommended to other communities wishing to reduce the euthanasia of healthy cats, increase save rates, lower cat impoundments, and reduce free roaming cat populations. There were substantial costs involved to perform the surgeries, and inject vaccinations and microchips on a massive scale. Communities must weigh the costs of SNR against future reduced cat handling in their shelters, and the desire to utilize non-lethal methods of cat control, to determine if this solution to the problem of too many cats entering their shelter is suitable in their area. This is a new concept in feral cat management. Results in the communities who have tried it all appear promising.

The establishment of this program involved a partnership between the shelter and non-profit volunteers. The collaboration has resulted in reduced cat euthanasia and impounds, with increased satisfaction of volunteer organizations advocating for more humane methods to handle the intact free roaming cat population.

This SNR program demonstrated that it did quickly reduce shelter cat intakes and shelter cat euthanasia above and beyond the traditional TNR program in San Jose and other areas. This program can also reduce future costs for animal control by reducing the shelter and free roaming cat populations. Further study on this effect seems warranted, along with another follow-up cat and dog population study.
